# Disease-Specific Expression of Conjunctiva Associated Lymphoid Tissue (CALT) in Mouse Models of Dry Eye Disease and Ocular Allergy

**DOI:** 10.3390/ijms21207514

**Published:** 2020-10-12

**Authors:** Philipp Steven, Sebastian Schwab, Anne Kiesewetter, Daniel R. Saban, Michael E. Stern, Uta Gehlsen

**Affiliations:** 1 Department of Ophthalmology, Faculty of Medicine and University Hospital, University of Cologne, 50924 Cologne, Germany; philipp.steven@uk-koeln.de (P.S.); anne@kiese-wetter.de (A.K.); michaelestern4@gmail.com (M.E.S.); 2 Division of Dry-Eye and ocular GvHD, Department of Ophthalmology, University of Cologne, 50924 Cologne, Germany; 3 Department of Internal Medicine I, Faculty of Medicine, University Bonn, 53127 Bonn, Germany; Sebastian.Schwab@ukbonn.de; 4 Institute of Experimental Immunology, Faculty of Medicine, University Bonn, 53127 Bonn, Germany; 5 Department of Ophthalmology, Duke University School of Medicine, Durham, NC 27701, USA; daniel.saban@duke.edu; 6 ImmunEyze, LLC., Irvine, CA 92606, USA

**Keywords:** conjunctiva-associated lymphoid tissue, CALT, antigen presentation, dry-eye disease, ocular allergy

## Abstract

Conjunctiva-associated tissue (CALT) is assumed to play a crucial role in the immune system of the ocular surface. Its function in several ocular surface diseases (OSD) is still not fully understood. This study investigates the function of CALT in mouse models of dry-eye disease and ocular allergy. Since antigen-presentation is the central similarity in the pathologies, this study focuses on antigen-presentation in CALT Morphology and the expression of CALT, which was investigated in mice after induction of dry-eye, ocular allergy, topical antigen-stimulation, and after local depletion of phagocytic cells. Antigen uptake was investigated after the application of fluorescent ovalbumin (OVA). OSD influences the appearance and morphology of CALT in a disease-dependent manner. Ocular allergy leads to an increase and dry-eye disease to a decrease in number and size of CALT. The development of CALT is dependent on the presence of APCs. Professional APCs are present in CALT, and soluble antigen is transported into the follicle. CALT appearance is disease-specific and indicative of differing functions. Although the specific involvement of CALT in OSD needs further study, the existence of functional APCS and antigen-uptake supports the hypothesis that CALT is an immunological key player at the ocular surface.

## 1. Introduction

Dry eye disease and ocular allergy are ocular surface inflammatory pathologies with high clinical relevance. Dry eye disease is a T-cell driven disease in which autoantigens (although still not fully identified) are discussed to play a significant role [1,2,3], whereas ocular allergy is a hypersensitivity reaction against widespread antigens known as allergens. Although the pathologies are different, antigen presentation is a key mechanism in the inflammation that develops in both diseases [4,5,6]. Antigen-presenting cells (APCs) with their ability to take up, process, and present antigens, in association with MHC II-molecules, play a key role in immunological defense and tolerance mechanisms. APCs are activated by inflammatory stimuli and generate local immune responses, e.g., in secondary lymphoid tissues, like mucosa-associated lymphoid tissue (MALT), to protect the body against antigens and to prevent pathological conditions. The conjunctiva presents immunological similarities with other mucosal tissues, and organized lymphoid follicles can be found as conjunctiva-associated lymphoid follicles (CALT) [7,8]. CALT is a regular component of healthy conjunctival tissue with an age-related expression, and several studies indicated its central role in the ocular surface immune system (reviewed in [9]). In mice, CALT develop exclusively in the nictitating membrane, but extensive histological studies described a similar morphology of CALT with B- and T-cells in the core, a lymphoepithelium containing M-cells, and high endothelial venules in humans and mice [7,8,10]. Furthermore, non-invasive in vivo two-photon microscopy revealed dynamic cellular processes and the ability to transport particles within the follicles [11]. 

Bacterial or viral infections can lead to hyperplasia of conjunctival follicles [12]. Moreover, biopsies of human conjunctivas revealed that in ocular allergy and dry eye disease, follicles change in number and size [13,14]. Additionally, hyperreflective cells were observed in confocal microscopy of CALT in patients with preservative-induced dry eye [9]. Morphological alteration of CALT was observed in patients with advanced stages of dry eye disease and discussed, leading to deregulation of CALT and contributing to chronic inflammation in dry eyes [14,15]. However, although the crucial role of CALT in inflammatory ocular surface diseases has been postulated, it is yet to be determined if a significant part of the central antigen presentation in these ocular surface diseases takes place in CALT or induces its assembly. Furthermore, modulations of the cellular structure of CALT itself by different inflammatory stimuli are to be expected. 

The goal of this study was to investigate a pathology-specific presence and morphology of CALT in mice, the analysis of professional APCs in this process, and their potential antigen-uptake and –presentation in CALT.

## 2. Results

### 2.1. Ocular Allergy and Dry Eye Disease Changes the Appearance of CALT in Mice

**Figure 1:**
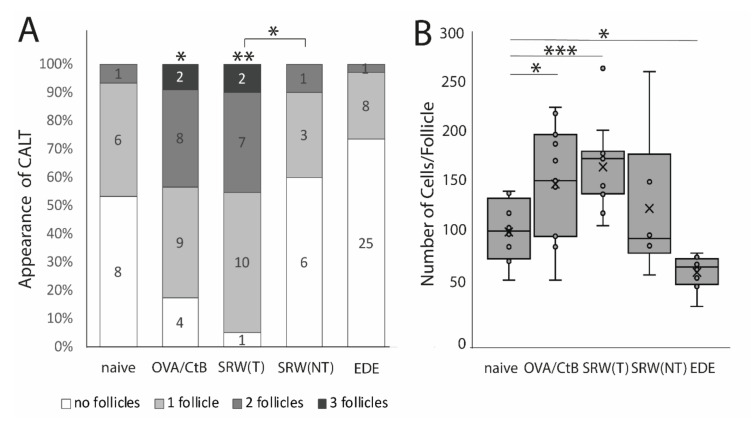
The appearance of conjunctiva-associated tissue (CALT). (**A**) Number of CALT in naïve (*n* = 8), OVA/CtB (ovalbumin/Choleratoxin B) stimulated (*n* = 12), short ragweed (SRW) allergy (T-treated; NT non-treated eye) (*n* = 15), and experimental dry-eye (EDE) (*n* = 17) mice. The number provided in the bars show the number of eyes investigated. Topical stimulation with OVA/CtB or SRW pollen leads to a significant increase in the number of follicles compared to naïve mice. Topically unchallenged eyes of immunized mice demonstrated significantly less CALT than stimulated SRW eyes, but no differences compared to naïve mice. (**B**) Average cell number per follicle. OVA/CtB and SRW mice had significantly bigger follicles compared to naïve. There was no difference between stimulated and non-stimulated SRW eyes (Kruskal-Wallis *p* = 0.0001, * *p* ≤ 0.05; ** *p* < 0.001; *** *p* < 0.0001).

**Figure 2:**
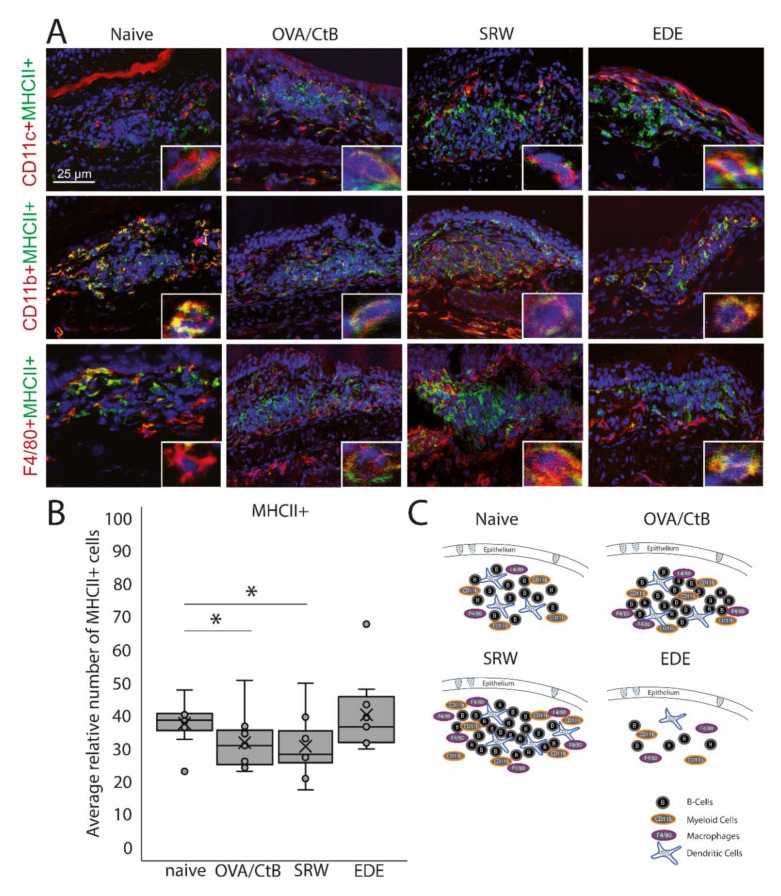
Antigen-presenting cells within CALT. (**A**) Immunohistochemistry of acetone-fixed cryosections. In naïve, OVA-CBt, SRW stimulated, and EDE mice CD11c+, CD11b+, and F4/80+ cells co-localized with MHC II+ were detectable. (bar = 25 µm; green: MHC II+; red: CD11c+, CD11b+, F4/80+; blue: nuclei). (**B**) The relative number of MHC II+ cells (in % of total cells per follicle). In OVA/CtB and SRW, the number of MHC II+ cells was decreased compared to naïve (Kruskal-Wallis *p* = 0.05, *p ≤ 0.05). (**C**) Schematic drawing of the cellular composition of CALT based on histological analysis.

Using several mouse models of ocular surface diseases, differences in the appearance of CALT were observed. Ocular SRW (short ragweed) allergy leads to an increase in the presence and the number of CALT follicles in the stimulated eyes, but not in the unchallenged control eyes, compared to naïve mice. Compared to OVA/CtB (ovalbumin/Choleratoxin B) stimulated CALT, no differences in the presence or the number of CALT per eye were detected (Figure 1A). Compared to naïve mice, stimulation with OVA/CtB or SRW, pollen both lead to a significant increase in the average size of CALT (Figure 1B). The size of CALT in unchallenged control eyes of SRW immunized mice was increased by trend compared to naïve CALT (*p* = 0.057). In contrast, EDE (experimental dry eye disease) did not change the presence of CALT compared to naïve mice, but resulted in a significant reduction of the size compared to naïve mice, but also SRW, and OVA/CtB treated mice (Figure 1B). After topical stimulation with SRW, follicles presented the highest density of APCs compared to naïve or unchallenged control eyes. In EDE eyes, only a few APCs were present in CALT (Figure 2C, schematic drawing of CALT based on detailed histological analyses).

### 2.2. Antigen-Presenting Cells in CALT

CD11b+, CD11c+, F4/80+ positive, and B-cells were identified in CALT histologically. CALT follicles were examined regarding the spatial organization of these cells. B-cells were organized in a central zone, whereas CD11c+ cells were randomly distributed within the B-cell zone. In the periphery F4/80+ and CD11b+ cells were present (Figure 2A,C). These cells revealed MHC II+ signals (Figure 2A). Although the number and size of CALT were increased in OVA/CtB and SRW stimulated eyes, no differences in the total number of MHC II+ cells were found compared to naïve mice. Compared to naïve mice, the relative number of MHC II+ cells (related to the total cell number per follicle) was significantly decreased after OVA/CtB and SRW stimulation (Figure 2B). 

**Figure 3:**
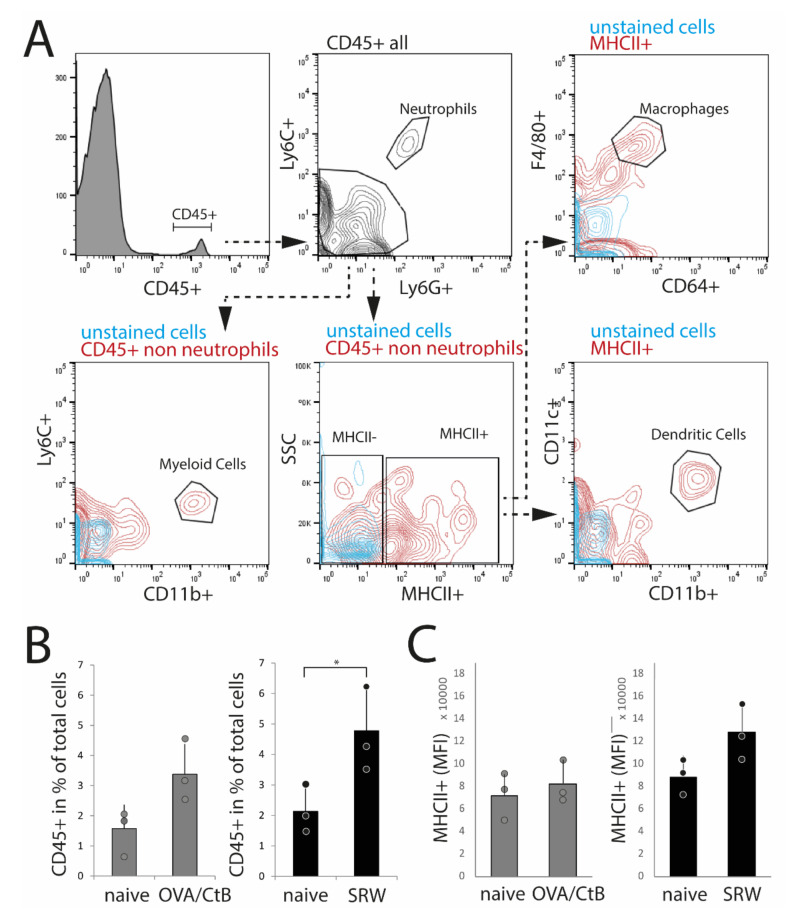
Flow cytometry analysis of isolated nictitating membrane tissues (CALT). (**A**) Gating strategy. (**B**) After SRW stimulation, the number of CD45+ cells was increased compared to naïve mice, reflecting the increased number and size of CALT. In OVA/CtB stimulated mice, the number of CD45+ cells was increased by trend (*p* = 0.07). (**C**) Median fluorescence intensity (MFI) of MHC II+ cells within the CD45+ non-neutrophils gate. No differences were found. (*n* = 3 independent experiments, *n* = 5 mice pooled/sample; * *p* < 0.05).

**Figure 4:**
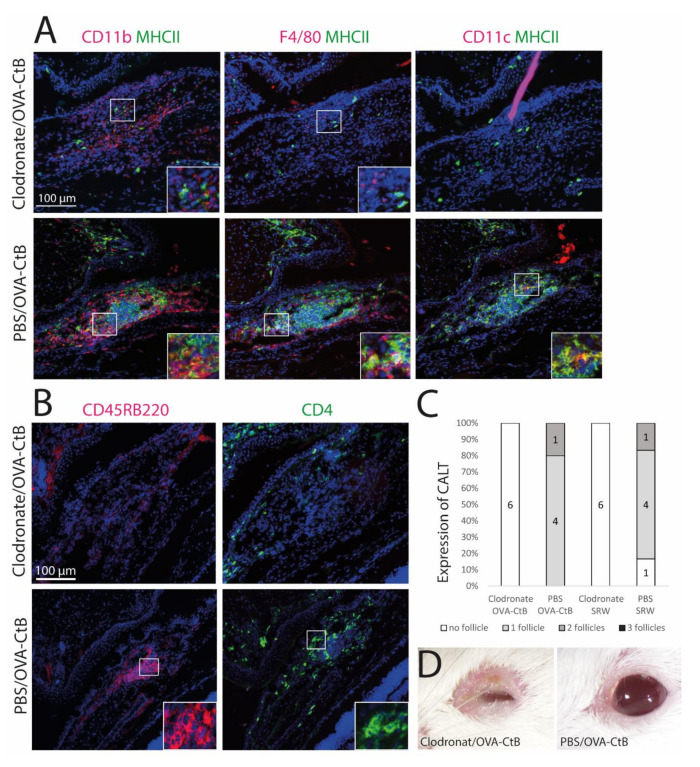
The development of CALT depends on the presence of APCs. (**A** + **B**) Immunohistochemistry of acetone-fixed cryosections (**A**): green: MHC II; red: CD11b, CD11c, F4/80; (**B**): green: CD45RB220 (B cells); red: CD4 (T cells); blue: nuclei; bar = 100 µm). After sub-conjunctival clodronate injections, only diffuse cellular infiltration without functional MHC II+ cells were present in the nictitating membrane (upper rows), whereas in PBS injected control mice, organized and functional CALT was present (lower rows). (**C**) The depletion of APCs by sub-conjunctival clodronate injection leads to a lack of CALT after OVA/CtB or SRW stimulation. (**D**) APC depleted mice suffered from severe blepharitis compared to PBS control mice, after OVA/CtB stimulation (8×/2 weeks).

To confirm histological results and cell characteristics, isolated nictitating membranes/CALT were analyzed by flow cytometry. Dead cells, doublets, and CD45-cells (non-leukocytes) were excluded from the analysis. Data were analyzed following the gating strategy, as presented in Figure 3A. In SRW stimulated mice, an increase of CD45+ leukocytes compared to naïve mice reflected the increase in expression and size of CALT, as seen in histology. In OVA/CtB stimulated mice, the number of CD45+ cells was trended towards an increase as compared to naïve mice (Figure 3B). Flow cytometry data further confirmed the general expression of dendritic cells, macrophage-like cells, and CD11b+ myeloid cells in CALT of naïve, SRW, and OVA/CtB stimulated mice. Neither the total number of MHC II+ APCs, nor the median fluorescence intensity (MFI) of MHC II+ cells was influenced by topical stimulation compared to naïve mice (Figure 4C). In further analysis, no differences in the amount of CD11b+ cells, macrophage-like, or dendritic cells in CALT were found (data not shown). No flow cytometry could be performed in EDE, since the number of relevant cells was below the detection range.

### 2.3. Antigen-Presenting Cells Are Crucial for the Development of CALT

Mice were treated with sub-conjunctival injections of clodronate liposomes to deplete conjunctival mononuclear phagocytes (MNPs) and subsequently stimulated with topical OVA/CtB or SRW. Clodronate induced depletion of the MNPs inhibited CALT formation, whereas control mice receiving PBS liposomes developed CALT in 100 % (Figure 4C). Mice treated with clodronate showed only sparse cellular infiltration without a spatial, defined structure of CALT. Few CD11b+, CD11c+, or F4/80+ cells were present, all without MHC II (Figure 4A,B: upper row). PBS (phosphate-buffered saline) injected control mice developed regular CALT follicles, with a distinct B-cell and T-cell zone (Figure 4B: lower row) and MHC II+ APCs (Figure 4A: lower row). Moreover, after CALT depletion, mice developed severe blepharitis after topical stimulation with OVA/CtB. PBS injected control mice treated with topical OVA/CtB were protected and maintained a healthy lid phenotype (Figure 4D). 

### 2.4. Antigen-Uptake and Presentation in CALT

**Figure 5:**
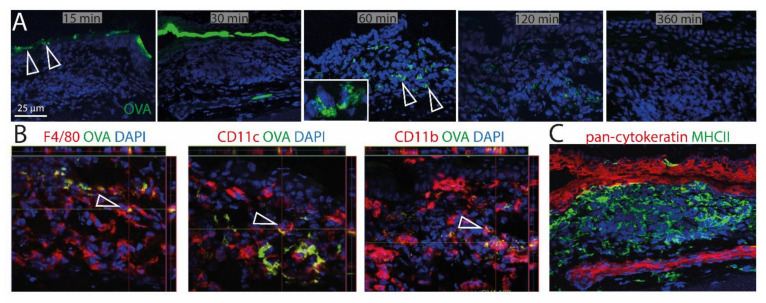
Uptake of soluble antigen in CALT. (**A**) Upper row. Topically applied, fluorescent OVA was transported via the lymphoepithelium into KLH/CtB induced CALT. OVA was detectable 60–120 min after application within CALT (see inlay). Three hundred and sixty minutes after the application, OVA was no longer detectable. Arrows show OVA accumulated above and within CALT. (**B**) Image stacks proved a co-localization of F4/80+, CD11b+, CD11c+ cells in CALT with OVA. Arrows indicate OVA uptake by APCs in CALT. (**C**) Epithelial cells above CALT do not express MHCII (green). (green: OVA; red: CD11b, CD11c, F4/80, pan-cytokeratin; blue-nuclei; bar = 25 µm).

To investigate the ability of CALT to uptake soluble and particulate antigen and a possible presentation by APCs, fluorescent OVA was applied to the ocular surface of KLH/CtB (keyhole limped hemocyanin/Choleratoxin B) stimulated mice. Fluorescent OVA was detected as a gradient constructed predominantly above the lymphoepithelium 15–30 min after application (Figure 5A). 60 min after application, OVA was detectable within CALT, 120 min after application, the visual quantity of OVA was declined, and 360 min after the application antigen was no longer detectable in CALT. Furthermore, a co-localization of OVA with CD11b+, CD11c+, as well as F4/80+ cells, was demonstrated (Figure 5B). Epithelial cells did not express MHC II (Figure 5C).

## 3. Discussion

CALT is a physiological component of the healthy ocular surface and appears under optical examination as a conjunctival follicle. Inflammatory ocular surface diseases change the number and size of CALT follicles in humans [9], which was also demonstrated in this study. In ocular allergy, a significant increase of CALT, together with bigger follicles was detected. The reason for this is the infiltration of APCs and the development of a massive B-cell zone in CALT after SRW stimulation. Conjunctival CD11b+ cells are described as crucial in the development of ocular allergy [5,6], which explains the increase of these cells in CALT. It is also known that B-cell-derived plasma cells are found preferably in the germinal centers of lymphatic tissues (like CALT) in the context of allergy [16]. In contrast, EDE leads to a decrease of CALT in this study. This is contrary to published results investigating human biopsies from patients with advanced dry eye disease [17]. This may be because patients, in contrast to the controlled environmental conditions of experimental models, are exposed to various risk factors and different antigens every day and may suffer from additional diseases (e.g., ocular allergy). In patients, the ocular surface challenge during dry eye disease is a complex process. Therefore, the increased presence of the follicles in patients with dry eye disease might be un-related to the pathology itself, and it has to be considered that the follicles are not pathognomonic for dry eye disease.

Mucosa-associated lymphoid tissues (MALT) are described to be both, effector, as well as inductive sites of the adaptive immune system. The development of follicles and a subsequent antigen presentation occurs in intestinal MALT to protect the body, e.g., after microbial infections and to prevent a systemic antigen uptake, and thus, infection or sepsis [18]. However, the development of MALT can also maintain the inflammatory response in autoimmune disorders [19]. The existence of regulatory T-cells in CALT, as shown previously by our group [10], also supports both immunosuppressive and immunoregulatory mechanisms in CALT. Therefore, the pathology-specific differences in CALT expression might be due to the diverse functions of CALT in different diseases. Another possible explanation for the decrease of CALT in EDE could be systemically applied scopolamine. Scopolamine leads to an inhibition of acetylcholine-mediated stimulation by blocking muscarinic acetylcholine receptors. This leads to a reduction of tear secretion, but also an increase of CD4+, CD11b+, and CD11c+ cells in the lacrimal glands [20]. On the other hand, scopolamine can also result in immunological inhibition of leukocyte infiltration [21]. Furthermore, different mouse strains are used to induce ocular allergy or EDE. It is known that, for example, Balb/c and C57BL/6 mice present strain-related differences in the pro-inflammatory cytokine profile of the ocular surface [22]. Own unpublished observations also indicate less frequent follicles in C57BL/6 mice compared to Balb/c (see Figure S1). This is similar to the MALT of the gut. Here, C57BL/6 mice were also shown to express fewer and smaller follicles compared to Balb/c mice [23,24]. In conclusion, the reason for the reduction of CALT in the context of EDE in this study remains unclear.

The study presented investigated APCs in CALT in the context of the most common chronic ocular surface diseases: ocular allergy, and dry-eye. The results of the study demonstrated the existence of different and functional APCs within murine CALT. Clodronate injections erased CALT and further proved the existence of APCs to be a key factor for follicle formation and maintenance. The spatial organization of the APCs in CALT follicles thereby was congruent to other MALT. B-cells reside in the center of the follicle in MALT and form a B-cell zone [25]. Such an arrangement of B-cells in CALT was described previously in humans [7] and mice [10], and was also found in this study. The existence of a B-cell-zone was demonstrated for all examined animal models except for EDE. 

CD11c, CD11b, and F4/80 positive cells were detected in CALT. CD11c is commonly considered as one marker of dendritic cells (DCs), whereas cells with a high expression of F4/80 are regarded as macrophages. CD11b is a universal surface marker, unmasking myeloid cells, including monocytes and macrophages [26], as well as dendritic cells [6]. A limitation of the study was that the analysis of classic APCs in CALT based on immunohistochemistry was complicated by the fact that most APC types and -subtypes share expression of common surface markers on the one hand and are characterized by a range of markers with different expression levels on the other hand [27]. Therefore, a definite association of histologically detected cells to a certain cell population was difficult. Moreover, flow cytometry of CALT was challenging because of the extremely low number of relevant cells. Especially in EDE, the number of cells was too low to be further analyzed. However, taking the established spatial arrangement of the APCs in other MALT and the immunohistochemical results of this project into account, an interpretation of the detected cells to a cell type in CALT was possible.

The organization of APCs in CALT appears to be particularly useful upon consideration of the individual APC function. DCs are considered the most potent stimulators of T-cells, among other things, due to the fact that MHCII complexes occur more often on mature DCs compared to other APCs [28,29]. Therefore, the localization mainly in the T-cell zone of CALT appears as a logical consequence, since there may occur a direct and efficient T-cell stimulation. Macrophages are characterized by a high phagocytotic potential, but a lower MHCII expression and ability for antigen presentation compared to DCs [26]. Depending on their M1 or M2 phenotype, they are involved in steady-state tissue homeostasis, the induction or resolution of inflammation, and tissue remodeling. In other secondary lymphoid organs, the most prominent macrophage subsets are CD169+ (Siglec1) macrophages mainly involved in immune regulation, but also antigen presentation to B-cells [30]. In CALT, macrophages were present predominantly outside the T- and B-cell zone, and no Siglec1 positive cells were detected. Comparably, macrophages were also considered to be not present in the germinal centers of Peyer’s Patches (PP) in the gut [31]. Only very recently, it was shown that this was only due to the lack of specific markers for these unique, atypical macrophage populations [32]. Macrophages in the PP lack “classical” macrophage marker like F4/80, CD169, MMR/CD206, or FcGR1/CD64. Depending on the localization, and due to local variation of the microenvironment, different sub-populations of macrophages with different functions, gene expression profiles, and phenotypes exist in the PP. It is possible that different macrophage populations might also exist within CALT, but could not be detected so far. 

Most APCs in CALT expressed MHC II, such they can be considered as activated. However, also MHC II negative APCs were identified in CALT. It could be hypothesized that immature, tolerogenic APCs would be represented by MHC II negative, but the activated, immunogenic APCs by MHC II positive cells. Thereby a balanced state between immunity and tolerance would be ensured. This would guarantee a reliable immune response, but also prevention of excessive inflammation. This, in turn, enables immunoregulation in CALT in terms of peripheral tolerance. Experimental depletion of CALT in this study then resulted in disbalance and loss of tolerance leading to severe blepharitis. In contrast, animals with intact CALT follicles were protected against such local inflammation after antigen stimulation. Moreover, immunoregulatory or immunosuppressive mechanisms most likely take place as CD4+CD25+FoxP3+ regulatory T-cells in CALT has been shown previously by our group [10]. Overall, through co-existence of such immune inductive and regulatory mechanisms, CALT is seen as an immunoregulating site of the mucosa comparable to MALT in other mucosal surfaces. 

The ocular surface is constantly exposed to antigens. To avoid excessive inflammation and tissue damage, immune tolerance mechanisms are crucial. Goblet cell-associated passage of antigens through the conjunctiva and controlled processing plays a crucial role in the induction of the local immune tolerance, but is disturbed in dry eye disease and age-related ocular surface inflammation [33,34]. Previously we demonstrated that particles and bacteria are also directly transported into CALT [10,11]. This study further demonstrated that uptake and presentation of soluble antigen take place in CALT. Soluble ovalbumin could be detected 60 min after topical application within the follicles co-localized to APCs. This is congruent with the results of [35,36], who demonstrated phagocytosis of antigens into CALT in guinea pigs and turkeys between 5-60 min after application. [36] also proved an accumulation of antigen over time. To induce an immune response in MALT, the present antigen has to penetrate the epithelial barrier and entrance the follicles. Epithelial cells can be involved in antigen uptake, and an expression of MHCII by intestinal epithelial cells was described in humans, rats, and mice [37]. The transport of ovalbumin by epithelial cells has been demonstrated in the intestine. Here the antigen was detectable as soon as 10 min after exposure [38]. This suggests that epithelial cells, particularly under pathological conditions, can act as APCs. In this study, however, no antigen-presenting properties of epithelial cells above CALT were found. CALT forms a lymphoepithelium containing cells with ultrastructural properties of M-cells, which transport antigen across the barrier [10,39,40]. Fluorescent-labeled antigen could then be visualized in APCs located directly below the epithelial barrier in CALT. Although the presented data does not provide a mechanism of how the antigen is processed and which exact immune response follows antigen exposure within CALT, we conclude that the APCs within CALT contain antigen to either induce a local immune response or carry antigens into the draining lymph node. As fluorescent-labeled ovalbumin within CALT was absent as early as two to six hours after application, this leads to the conclusion of drainage of soluble antigen or antigen-containing APCs to the lymph node.

In conclusion, the results presented support the hypothesis that CALT is a key player in the immunology of the ocular surface, both for the initiation and regulation of immune response. Murine CALT was described as an effective vaccine delivery route, protecting mice by the increase of specific IgA antibodies in the mucosa, but also systemic IgG production against lethal viral or bacterial infections [40]. We, therefore, suggest including CALT into current concepts of the ocular surface disease, in particular, the understanding of afferent and efferent immune responses. Furthermore, the results of the basic research study presented will form a basis for more detailed studies aiming at antigen-presentation in CALT as a potential treatment option. Current topical, therapeutic approaches mostly aim to treat already existing inflammation, e.g., by targeting the T-cell-driven response in dry eye disease [41,42]. However, the induction of the inflammation takes place at a distinct earlier time point, starting with the presentation of antigen. Using substances that influence the antigen-presentation in CALT at the very beginning might decrease the severity of the inflammation. 

Depending on the disease, targeting antigen-presentation could be used as a preventive approach regarding the exacerbation of the disease. In dry eye disease, targeting of antigen-presentation could prevent the repeated generation of autoreactive T-cells, which trigger sequelae of inflammation, often trough phases of increased desiccation or eye surgery. For ocular allergy, in particular, seasonal forms, the disruption of allergen processing through APCs could reduce disease activity.

For this, more detailed studies are needed, to better understand the disease-, time- and antigen-depending role of specific APC subtypes in CALT in ocular surface diseases. Furthermore, nothing is known about the interplay between the APCs (namely DCs) in CALT, in MALT of the tear ducts and nasopharynx, and the regional lymph nodes. Also, the role of co-stimulatory molecules (B7.1 or B7.2) for the effective antigen-presentation in CALT needs to be investigated, e.g., by using knockout mouse models. 

## 4. Methods

### 4.1. Animal Experiments

Female Balb/c and C57BL/6 were purchased from Charles River Laboratories (Sulzfeld, Germany) and housed under standard conditions. The mice were screened for ocular abnormalities (corneal opacity, scarring, lid edema, etc.) before the experiments. No abnormalities were observed. No animals were excluded from the study, and no unexpected deaths of animals occurred during the study. The treatment of the animals was undertaken following the regulations of the University of Cologne and the local committees of North Rhine-Westphalia (Germany). All procedures were approved by the German animal welfare authorities (approval code: 84-02.04.2013.A098, approval date: 18.11.2013) and were performed following the ARVO statement for the use of animals in ophthalmic and vision research. 

Ocular allergy was induced in Balb/c as published, using short ragweed pollen (SRW, Hollister Stier, Spokane, DC, USA) [43]. Mice were immunized i.p. with 50 µg SRW in Imject^®^ alum adjuvant (Thermo Fisher Scientific, Schwerte, Germany) and PBS. Ocular allergy was induced in the right eyes by topical administrations of 1.5 mg SRW in 10 µl PBS per eye for seven consecutive days. The left eye remained as a control without topical allergen stimulation. Mice were examined 20 min after each SRW application for symptoms of conjunctivitis (tearing, lid edema, conjunctival vasodilatation, chemosis). 

Experimental dry eye disease (EDE) was induced using a (modified) desiccating stress protocol, as previously published [44,45]. C57BL/6 mice were exposed to reduced humidity (<25%) and a constant airflow for 14 days. Additionally, scopolamine (Sigma Aldrich, Germany) was administered subcutaneously using Alzet^®^ pumps (0.7 mg in 110 µL PBS).

Non-pathological CALT was stimulated by the topical application of either Ovalbumin (OVA, Sigma Aldrich, Germany) or keyhole limpet hemocyanin (KLH, Calbiochem^®^Merck, Germany) together with Choleratoxin B (CtB, Sigma Aldrich, Taufkirchen, Germany) at eight times as previously published [10,11]. Naïve mice were used as a control. To track the up-take of soluble antigen in CALT, 5 µL of fluorescent-labeled OVA (Thermo Fisher Scientific, Schwerte, Germany) was applied topically to anesthetized mice with OVA/CtB stimulated CALT. Mice were sacrificed 15, 30, 60, 120, and 360 min after application. Whole eyes, including the nictitating membranes, were dissected and fresh frozen in liquid nitrogen for further analysis. 

To deplete mononuclear phagocytes (MNPs), mice were treated with two sub-conjunctival injections of clodronate-loaded liposomes (Clodrosome^®^, Encapsula NanoSciences LLC, TN, USA) before topical OVA/CtB or SRW stimulation. Control mice received PBS containing liposomes. Injections were given under short-term narcosis with Ketamine (Ketanest^®^S, Pfizer, Berlin, Germany) /Xylazine (Rompun^®^, Bayer Health Care, Leverkusen, Germany). During the injection, the eye was additionally anesthetized with a drop of Conjuncain EDO^®^ (Bausch & Lomb, Heidelberg, Germany).

### 4.2. Immunohistochemistry

Fresh frozen tissue was acetone-fixed and sectioned using a Leica CM3050 Cryostat (Leica, Germany). Sections (8–12 µm) were incubated with antibodies according to the manufacturer’s instructions. The specific antibodies used for immunocytochemistry are described in detail in Table S1 of the supplemental part of the manuscript. For all antibodies, positive control stainings (primary and secondary antibody) and negative control stainings (secondary antibody only) using either tissue sections of lymph nodes (cytokeratin, CD4, CD8) spleen (MHCII, LYVE1, CD11b, CD11c, CD45RB220) or Peyer’s patches (F40/80) were carried out. Nuclei were stained with Hoechst-dye or DAPI. Images were recorded with a Zeiss LSM Metaconfocal 510 (Zeiss, Jena, Germany) confocal microscope and analyzed using Zeiss LSM Image Examiner (version 3.5) and ImageJ (1.48v) software containing the “cell counter“ plugin. 

To prove the existence of defined CALT follicles, sections were analyzed for the expression of CD4+, CD8+ T-cells, B-cells, and surrounding blood- and lymph vessels according to the definition published previously [11,39] (Figure S2). Only follicles with defined characteristics of CALT were included in further histological analysis. To estimate the size of CALT, the total number of cells was calculated. The number of all cells was added, and the result was divided by the number of slides analyzed. For the calculation of specific APCs, the most central section of each follicle was chosen. The number of APC was related to the average total cell number of the follicle. To compare CALT in the different models, the number of antigen-presenting cells (APC) was related to the total cell number of the follicles.

### 4.3. Flow Cytometry

Nictitating membranes of naïve, OVA stimulated, and allergic mice were dissected. Because of the small amount of tissue, the eyes of five mice each were pooled into one sample for analysis. Tissues were digested with 2 mg/mL collagenase D and 0.1 mg/mL DNAse I (Roche, Germany) in HBSS. Cells were transferred to FACS buffer (0.5% BSA, 1% FBS, EDTA 1:50 in PBS), and blocked with Fc block anti-mouse CD16/32 (eBioscience, Germany). Cells suspensions were incubated with CD11b, CD11c, Ly6G, Ly6C, CD45, CD64 (BioLegend, Germany), MHCII, SiglecF (BD Bioscience, Germany), and F4/80 (eBioscience, Germany) antibodies according to the manufacturer’s instructions for 30 min on ice and protected from light. After washing the cells, the pellet was resuspended in PBS, and eFluor450 fixable viability dye staining was performed (BD Bioscience, Germany). Cells were then washed and fixed in stabilizing fixative (BD Bioscience, Germany). FACS analyses were performed using either an LSR Fortessa (BD Bioscience) or Guava easyCyte (Merck Millipore) cytometer. Raw data were analyzed using FlowJo software (FlowJo LLC, Ashland, OR, USA). Three independent experiments were conducted.

### 4.4. Statistics

Data were tested for Gaussian distribution and equality of the variances using the Kolmogorov-Smirnov and Levene test. Depending on the results, either one-way ANOVA (post hoc test: least significant difference LSD) for multiple comparisons or the Student’s t-test and Welsh’s-test for two groups, were used to analyze statistical differences. A *p*-value < 0.05 was considered to be significant. All statistical analyses were performed using SPSS (vs.25) software (IBM Corp., Armonk, NY, USA).

## Supplementary Materials

Supplementary materials can be found at https://www.mdpi.com/1422-0067/21/20/7514/s1.

## Abbreviations

APC	Antigen-presenting cell
CALT	Conjunctiva-associated lymphoid tissue
CtB	Choleratoxin B
EDE	Experimental Dry-Eye
MALT	Mucosa-associated lymphoid tissue
MHC	Major-histocompatibility complex
MFI	Mean fluorescence intensity
OSD	Ocular surface disease
OVA	Ovalbumine
PBS	Phosphate-buffered saline
SRW	Short ragweed
