# Supplementary material for: Disease-Specific Expression of Conjunctiva Associated Lymphoid Tissue (CALT) in Mouse Models of Dry Eye Disease and Ocular Allergy
Source: Int J Mol Sci. 2020 Oct 12;21(20):7514. doi: 10.3390/ijms21207514 (PMC7589149; doi:10.3390/ijms21207514)
Supplement: Supplementary file 1 [file ijms-21-07514-s001.pdf]

Supplemental information

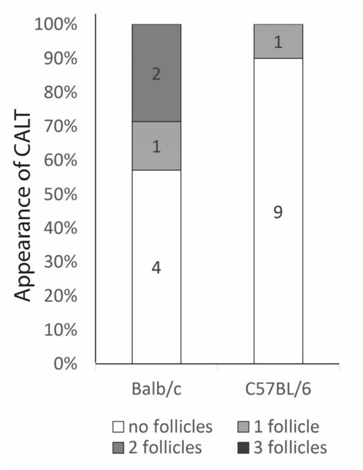

Supplemental Figure S1: Appearance of CALT in naïve Balb/C and naïve C57BL/6 mice.

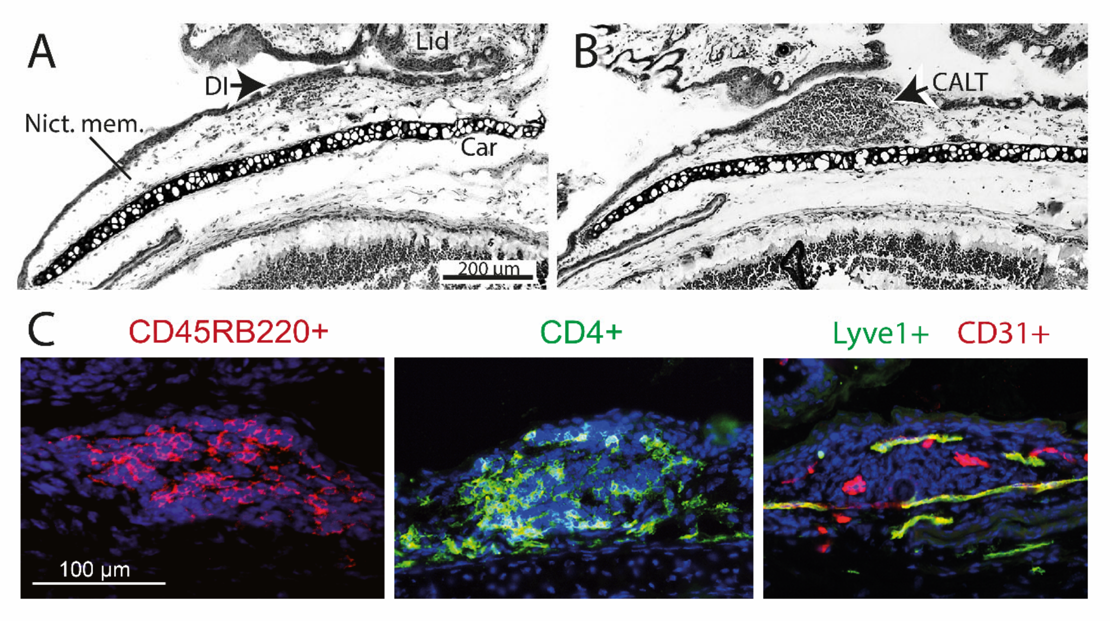

Supplemental Figure S2: Representative images depicting the criteria for the definition of CALT. In contrast to (A) diffuse cellular infiltrations, (B) follicles are defined as condensed accumulation of cells, containing a central B- cell and T cell zone, with surrounding blood vessels and lymphatics. **A+B)** Nictitating membrane in OVA/CtB stimulated mice (toluidine staining). Outside the CALT follicles only few cells are present. For FACS only the nictitating membrane tissue was harvested, digested and further analyzed. **C)** Immunohistochemistry of a CALT follicle show CD45RB220+ B cells (red), CD4+ T cells (green) and adjacent blood- (red) and lymph- (green) vessels. (Nict.mem.= nictitating membrane, Car = cartilage, DI = diffuse cellular infiltration).

Supplemental Table S1: Antibody-panel used for immunohistology.

Antibodies (clone)	Target	Manufacturer	Catalog-no.
CD11c (N418)	Dendritic Cells	Acris Antibodies	AM05514PU-L
F4/80 (A3-1)	Macrophages	Invitrogen	MA5.16630
CD45RB220 (RA3-6B2)	B-Cells	BioLegend	103201
CD11b (M1/70)	Monocytes	Acris Antibodies	SM014A
Pan-cytokeratin	Epithelial Cells	Novus Biologicals	NB600-579
CD4 (GK1.5), CD8 (53-6.7)	T-Cells	eBioscience	14-0041; 14-0081
CD31 (MEC13.3)	Blood vessels	BD Bioscience	553371
Lyve1	Lymphatic vessels	Acris Antibodies	DP3513
MHC II (OX-6)	APC	BioOrbyt	orb44830
DAPI	Nuclei	Sigma Aldrich	D9542
